# Identifying avian influenza hotspots in wild birds in the Netherlands

**DOI:** 10.1371/journal.pone.0341829

**Published:** 2026-02-12

**Authors:** Ronald Petie, Eduardo de Freitas Costa, Christian Kampichler, Roy Slaterus, Jose L. Gonzales

**Affiliations:** 1 Department of Epidemiology, Bioinformatics and Animal Studies, Wageningen Bioveterinary Research, The Netherlands; 2 Sovon, Dutch Center for Field Ornithology, Nijmegen, The Netherlands; Central Laboratory for Evaluation of Veterinary Biologics, Agricultural Research Center, EGYPT

## Abstract

Highly Pathogenic Avian Influenza (HPAI) threatens wild and domestic birds, mammals, and humans. The global spread of HPAI through wild birds requires timely, spatially accurate detection for enhanced preventive measures. However, detecting outbreaks in wildlife is challenging due to reliance on opportunistic sampling and testing of small numbers of wild animals, resulting in insufficient data on the temporal and geographical distribution of infections. We aimed to create a HPAI hotspot prediction model for the Netherlands, using wild bird mortality reports and confirmed HPAI incidents from 2016–2022. Variables for human, wild bird, and sampling density were used as statistical adjusters in various combinations. The Bayesian binomial model employed a case-crossover design, where HPAI incidents in wild birds were cases, and controls used the same date and location as the case but transposed to 2019, when no HPAI was reported in the Netherlands. We tested 24 models in a ten-fold cross-validation design. The best model had an area under the curve score of 0.68, a sensitivity of 0.47, and a specificity of 0.79, and included wild bird mortality and density. The yearly spatial distribution of predicted wild bird HPAI outbreak areas generally matched laboratory-confirmed HPAI cases, especially from 2020 onward, except for an atypically intensively sampled area west of Amsterdam before 2020. Most HPAI outbreak predictions occurred from October through March. Our model highlights potential HPAI outbreak areas over time and can be used to direct sampling efforts, potentially increasing both effectiveness and timeliness.

## Introduction

Highly Pathogenic Avian Influenza (HPAI) is a severe and highly contagious viral disease that poses a significant threat to wild bird populations. In the Netherlands, the risk is particularly acute due to the country’s wetlands and coastal areas, which serve as crucial stopover points for migratory waterbirds which are, along with other species using major migration routes, the main reservoir of the avian influenza virus. Migratory waterbirds are often found in dense flocks, creating ideal conditions for the virus to spread [1] and cause large-scale outbreaks.

Waterfowl, such as ducks and geese, are natural carriers of avian influenza viruses, with some species being able to carry the virus without showing severe symptoms. This enables the virus to spread over vast distances during migration [2,3]. Since its first significant intercontinental spread in 2014, the H5Nx HPAI viruses of the clade 2.3.4.4 have been a recurring problem, causing substantial mortality among wild birds and posing risks to domestic flocks [4–6].

Several European countries have implemented HPAI surveillance in wild birds as an early warning system for the potential spread of the virus to poultry [7]. In the Netherlands and across the European Union, surveillance mainly relies on public reports of wild bird mortality, submitted by the general public, bird watchers, and wildlife professionals. These community members play a crucial role in reporting unusual bird deaths or abnormal behaviour, which can indicate the presence of HPAI and prompt the submission of samples for laboratory confirmation. This system, known as “passive surveillance” [7], extends the reach of monitoring beyond what professional agencies alone can achieve. However, this reliance on public reporting and sample submission introduces biases in disease occurrence data, affecting time, location, and species coverage. Some notable biases include:

**1. Species visibility**: Larger or more visually striking species, such as swans (genus *Cygnus*), are more likely to be noticed and reported than smaller species, such as wigeons (genus *Mareca*). The same is true for species living in open, accessible landscapes.
**2. Species susceptibility**: species with higher susceptibility, tend to show severe symptoms or experience higher mortality and are more likely to be detected, while less affected species may go unnoticed [3]. Susceptibility may even vary within the same species across different years [8,9].
**3. Population density**: More abundant species, especially those living in dense groups, are more likely to be reported, as higher population densities increase the chance of detection.
**4. Monitoring strategy**: In the Netherlands, a decision was made to refrain from testing individuals of a species in a given region, if that species recently had a confirmed positive there. This approach, while cost-effective, may introduce temporal bias in disease distribution by leaving later mortalities unreported.

To address these biases, identifying high-risk areas for sampling and testing at different times of the year could improve surveillance accuracy. Additionally, (increased) active surveillance could be considered to enhance timeliness and reduce bias. Active surveillance involves the systematic sampling of live birds. In addition, (intensified) international collaboration could further improve species coverage. By sharing data, countries can identify more susceptible wild bird species than those recognized through national efforts alone [7,10].

As HPAI continues to threaten wild birds [11,12], wild mammals [2,13–15], and poultry, maintaining robust surveillance systems remains a priority. The effectiveness of HPAI surveillance depends on several factors, including geographical and species coverage, the timeliness of detection, and the accuracy of reporting and diagnostic methods. Risk-based surveillance, whether passive or active, that targets high-risk areas (“hotspots”) could help mitigate the biases mentioned above and improve overall effectiveness.

This paper presents a method for identifying potential hotspots for HPAI case fatalities by analysing bird mortalities reported to Sovon (the Dutch Centre for Field Ornithology) and HPAI incidents in both domestic and wild animals, as documented by the World Organisation for Animal Health (WOAH).

## Materials and methods

Our main question was how to determine if wild bird deaths reported by volunteers were due to HPAI infection. We hypothesized that two main factors could indicate an ongoing HPAI outbreak.

1. An increase in wild bird deaths in the area where a dead bird was reported.
2. Proximity in time and location to a laboratory-confirmed HPAI case.

The first factor is meant to indicate general disease spread, while the second confirms it is HPAI. Other factors that might affect the number and spatial distribution of reports of bird mortality include wild bird density, human population density, and sampling density. Higher bird density likely means higher mortality during an outbreak and a higher baseline mortality in the absence of disease. Human population density affects where reports come from.

In this study we stayed close to the wild bird data, in order to show the value of volunteer data on its and keep the results interpretable. Other variables could be added to later iterations of the model to incorporate processes like weather and bird migration. Habitat suitability is already implicitly part of the model as it was used to model bird densities (S2 Text).

### Study design

Results were obtained in two steps. First, to develop the model, we applied a case-crossover design to the HPAI incident data. This gave us cases and controls that we used for calculating the model performance measures that guided the development of the model. Second, when a satisfactory model was found, it was applied to the dataset with wild bird mortalities to predict HPAI infection probability for each wild bird mortality and answer our main question: which wild bird deaths were likely due to HPAI infection?

In the case-crossover design, each HPAI outbreak incident (n = 982) between 2016 and 2022 served both as a case and its own control by shifting the incident to the same day and month in 2019, a year with no reported HPAI outbreaks in the Netherlands, despite ongoing testing (Table 1), ensuring likely HPAI virus absence.

**Table 1:** Wild bird mortalities tested for HPAI in the Netherlands per year, as a part of the passive surveillance program.

2016	2017	2018	2019	2020	2021	2022
505	601	710	659	854	1103	1926

### Data

**Fig 1:**
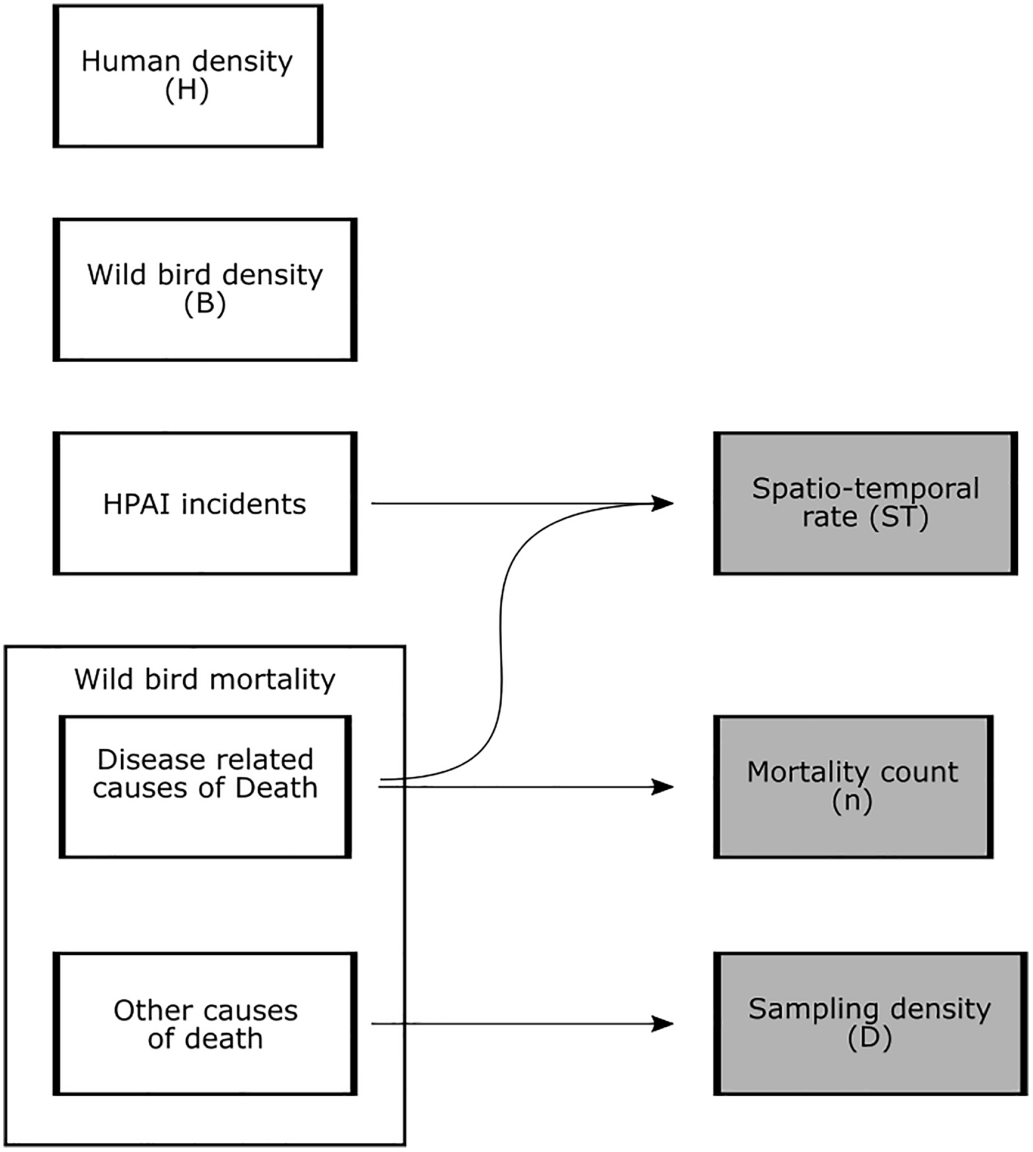
Overview of primary data and processed data. White boxes represent primary data, while grey boxes represent processed data. If a variable is used in the analysis, its abbreviation is indicated within parentheses.

For this study, we used two types of data:

1. Primary data, which were directly collected from the relevant databases and described in Table 2, and
2. Processed data, generated by processing the primary data as outlined in Fig 1.

**Table 2:** Description of primary data sources.

Data	Source	Inclusion criteria	Temporal scale	Spatial	
				Original	Converted
Human density	Statistics Netherlands	Number of residents.	2022	100m x 100m	5km x 5 km
Wild bird density	Sovon	190 HPAI susceptible species.	2014-2016	5km x 5 km	–
HPAI incidents	WOAH	HPAI incidents in poultry and wild birds.	1-1-2014–31-3-2023	Point observations	–
Wild bird mortality	Sovon	Mortality in 190 HPAI susceptible species, where the animal had died within 48 hours of discovery. Only land-based observations within the borders of the Netherlands were kept, with a buffer zone of 1 km.	1-1-2016 to 31-12-2022	Point observations	–

Sovon, Dutch Centre for Field Ornithology; WOAH, World Organisation for Animal Health. Note that WOAH HPAI incidents for 2023 were included for calculation of the spatiotemporal distance. See text for details.

The primary dataset comprised four key data sources: human density, wild bird density, HPAI incidents and wild bird mortalities. Details regarding the sources and processing of these data can be found in Table 2 and are further elaborated in the sections below.

### Primary data

Data on human population density for the year 2022 was downloaded from the Dutch statistics bureau (CBS, [16]). In the time frame of our study, the human population density was considered to be static, especially when taking into account that the model uses the log_10_ of the original population density value.

Data from Sovon consisted of two datasets: wild bird abundance gridded at 5 by 5 kilometres and wild bird mortality reports. Both rely on volunteers for data collection and both were filtered to included only 190 bird species [17] sensitive to HPAI infection (see Table A in S1 File). Modelled wild bird abundance in winter was taken as wild bird density.

The wild bird abundance model was based on various bird monitoring schemes, including: the Waterfowl Monitoring Network [18], Transect-Point Counts [19], Bird Atlas of the Netherlands [20], LiveAtlas [21] and the roosting monitoring network [18]. These schemes involve systematic counts and observations of bird species across different seasons and regions in the Netherlands. Data from these schemes were used in spatial modelling, employing techniques like regression-kriging [22] and random forests [23] to predict bird distribution and density. Environmental variables such as climate, habitat types, and land use are incorporated into the models to enhance accuracy. For more details on abundance modelling, see the S2 Text information on wild bird abundance modelling.

Wild bird mortality reports from 2016 to 2022 were analysed, again from the 190 species mentioned above. The reports were included only if the animal had died within 48 hours of discovery. Reports were assumed to be potentially disease-related when the cause of death was: ‘unknown’, ‘disease’, ‘water’ or ‘other’. Reports with causes of death ‘window’, ‘traffic’, ‘starvation’, ‘building’, ‘cat’, ‘wire’, ‘shot’, ‘rails’ and ‘windmill’ were used as indicators for the general reporting activity. Birds of multiple species reported on the same day and location were grouped and labelled as ‘multiple species’.

Some observations were located in the sea, a lake, or outside the country borders, which were considered mistakes and removed. However, in the tidal Wadden Sea, water levels can be so low that you can walk between the mainland and the islands, making it relatively easy to find bird mortalities offshore. Additionally, this region experienced high wild bird mortality. Therefore, we kept observations that were on land or at most 1 km offshore.

HPAI outbreaks were sourced from the World Animal Health Information System (WAHIS, [24]), filtered for the Netherlands and disease names:

1. *High pathogenicity avian influenza viruses (poultry) (Inf. with)* and
2. *Influenza A viruses of high pathogenicity (Inf. with) (non-poultry including wild birds) (2017-)*.

The data included outbreaks from 2014 to March 31, 2023, ensuring that wild bird mortalities reported in December 2022 had at least three months of corresponding HPAI outbreaks to be used for calculation of the spatio-temporal rate. All outbreak incidents were included, regardless of whether they involved poultry, wild birds or mammals. The outbreaks concerned the subtypes H5Nx, H5N1, H5N3, H5N4, H5N5, H5N6 and H5N8. In total, 79% of the outbreaks involved H5N1, 16% H5N8 and the remaining 5% were for the other subtypes. The viruses causing the outbreaks in 2014 were of the clade 2.3.4.4. Afterwards, all virus subtypes belonged to the clade 2.3.4.4.b.

### Processed data

Processed data consisted of: the density of the wild bird mortality sampling by volunteers, wild bird mortality counts and the spatio-temporal rate. These are explained in more detail below. Wild bird counts and spatio-temporal distances were calculated at the geographical location and date of each a) wild bird mortality, b) case incident from the case-crossover design and c) control incident from the case-crossover design. Note that for case-control pairs, the geographical locations were identical, but the dates were different.

The sampling density (Fig. 3E) included all wild bird mortalities with causes other than disease. Spatial interpolation of this data was performed using the SPARR package [25] with a fixed-bandwidth kernel of 13 km, determined using the Kelsall-Diggle method.

**Fig 2:**
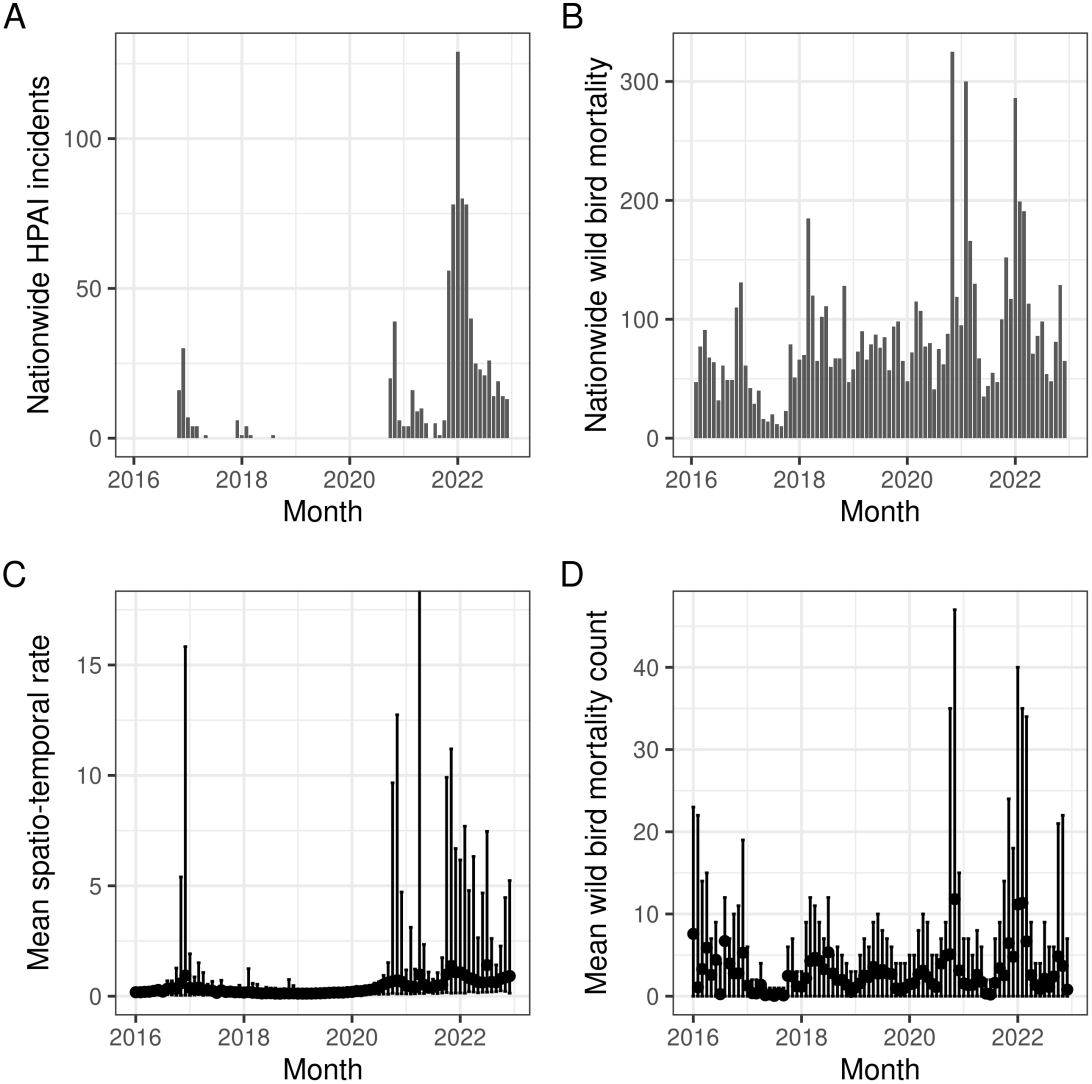
Monthly temporal Patterns in the primary and processed variables. The top panels display nationwide temporal patterns in primary data, while the bottom panels show processed data. **A)** Monthly nationwide counts of HPAI incidents in poultry and wild animals (including mammals). **B)** Nationwide reports of wild bird mortalities across 190 species of interest. Processed data was calculated for each wild bird mortality individually. **C)** The spatio-temporal rate measures the rate of HPAI incidents occurring in space and time around each wild bird mortality. **D)** Wild bird mortalities were also counted within a *±* 10-day window and a 25 km radius around each wild bird mortality. The bottom panels present monthly means and ranges, with filled circles representing the mean and bars indicating the minimum and maximum values.

Wild bird mortality was counted within increasing radii around each reported mortality and HPAI case, using a time window of 21 days, centred on the date of death (ten days before and ten days after the incident). We only included mortalities from 190 bird species that are associated with HPAI outbreaks. Guidelines for reporting suspicions of HPAI virus infections in the Netherlands state that you can report any wild bird mortality [26]. In order to keep the mortality reports with likely HPAI induced mortality we filtered for:

- Any observed mortality concerning *Anatidae* species,
- incidents where two or more different species were involved or,
- 20 or more individuals birds were reported dead for one species.

The motivation to filter for *Anatidae* species was their strong association with HPAI outbreaks, particularly in Europe before 2023 [7]. Next, when two or more species were reported or 20 or more individuals of the same species were detected, may indicate that a virulent strain of the virus is spreading.

Virus survival times on land can be several days to two weeks, depending on temperature, humidity, and surface type. Cooler temperatures and non-porous surfaces extend survival times [27]. In water, survival times are higher and the virus can persist for weeks to months in cold freshwater, while survival times decrease in warmer water and higher salinity levels [28]. For land and water combined, the survival times range from several days to months. The 21 day time window is a compromise. We tested the effect of extending the time window to 41, 61 and 81 days and found no significant change in the areas under the curve (AUC) of the developed statistical models (Table B in S1 Text). AUC is an evaluation metric for model performance which measures the ability of the model to discriminate between prediction classes.

Radii tested were 1, 2, 5, 10, 15, 20, and 25 km. The optimal radius was determined by calculating the Pearson correlation between the log_10_ of bird counts and the infection status, where cases were 1 and controls 0. We used training data for this, see the next chapter for more details on resampling of the data.

The spatio-temporal rates were based on spatio-temporal distances, which are a measure of the proximity to a confirmed HPAI case. For HPAI incidents, the distance to itself was excluded. The mean shortest spatial distance ($\mu_{D}$) between wild bird mortalities and HPAI incidents was 3.1 km, and the mean time difference ($\mu_{T}$) was 73.8 days. For each observation (i), spatio-temporal distances (STD) to HPAI incidents (j) were calculated as:


$$STD_{i,j}=\sqrt{D_{i,j}^{\text{2}}+T_{i,j}^{\text{2}}}$$

$D_{i}$ and $T_{i}$ are the scaled, unit-less distance measures below.


$$D_{i,j}=\frac{d_{i,j}}{\mu_{D}}$$

$$T_{i,j}=\frac{t_{i,j}}{\mu_{T}}$$

Here $d_{i,j}$ is geographical distance and $t_{i,j}$ the time difference between the wild bird mortality and the HPAI incidents. For each observation the lowest spatio-temporal distance was chosen. In the statistical model below, the spatio-temporal rate (ST=1/STD) was used instead of the spatio-temporal distance, because it provided a better fit to the data.Before usage in the statistical models, we took the log_10_ of all variables and standardised them (subtract mean and divide by standard deviation).

### Map boundary data

The maps in this manuscript were produced with the R package tmap [29] using the bundled dataset NLD_prov. According to the package documentation, NLD_prov is derived from data provided by Statistics Netherlands (CBS, [30]) and Kadaster Nederland [31], and publication is permitted provided the source is cited. The original boundary data are distributed as open government data (PDOK/CBS) and allow redistribution with attribution.

### Model building and validation

As previously mentioned, case and control data from the case-crossover design were used to inform model development. The dataset consisted of 982 cases and an equal number of controls, totalling 1964 observations. To assess model performance, we created a subset of the data to be used for training the models, containing 70% of the observations. The remaining 30% were reserved, and only used for testing the final performance of the model. The training data was resampled using 10-fold cross validation, into *analysis* and *assessment* subsets [32]. The *analysis* data was used to develop and train models, while the the *assessment* data was used to measure the performance during model development. To preserve data integrity, case-control pairs were kept together in each of the subsets.

We used a Bayesian binomial model, starting with the observed incident cases ($I_{i}$) arising from a Bernoulli process. We modelled HPAI infection probability of each observation (i) in the case-crossover design as logit-linear combinations of: the spatio-temporal rate (ST), wild bird mortality count (n), human density (H), sampling density (D) and wild bird density (B). The model was run in R version 4.2.2 using Rstan [33] through the rethinking package [34], using four parallel chains, with one thread per chain.

$I_{i}=Bernouilli\left(p_{i}\right)$

$logit\left(p_{i}\right)=\alpha +\beta_{S}ST_{i}+\beta_{N}n_{i}+\beta_{H}H_{i}+\beta_{D}D_{i}+\beta_{B}B_{i}$

As detailed in Table 3, twenty-four different models were tested. The models were built around the spatio-temporal rate (ST, b1-b8), wild bird mortality counts (n, c1-c8), or both (a1-a8) as a basis. All permutations of the static variables (human density, sampling density, and bird density) were tested.

**Table 3:** Models tested. Numbers indicate: 1, variable included; 0, variable excluded. Shading shows the grouping of the models based on the inclusion of *spatio-temporal rate* (*ST*) and wild bird mortality (n). The other variables are: human density (H), sampling density for wild bird mortalities (D) and wild bird density (B).

Model	a								b								c							
	1	2	3	4	5	6	7	8	1	2	3	4	5	6	7	8	1	2	3	4	5	6	7	8
ST	1	1	1	1	1	1	1	1	1	1	1	1	1	1	1	1	0	0	0	0	0	0	0	0
n	1	1	1	1	1	1	1	1	0	0	0	0	0	0	0	0	1	1	1	1	1	1	1	1
H	1	1	1	1	0	0	0	0	1	1	1	1	0	0	0	0	1	1	1	1	0	0	0	0
D	1	1	0	0	1	1	0	0	1	1	0	0	1	1	0	0	1	1	0	0	1	1	0	0
B	1	0	1	0	1	0	1	0	1	0	1	0	1	0	1	0	1	0	1	0	1	0	1	0

The posterior distributions from the models developed on the training subset of the data were used to make predictions of the probability that case, or control, observations were HPAI related ($p_{i}$). Next, optimal threshold values for dichotomisation of the data were determined using the cutpointr package [35]. The best performing model was selected based on the mean Area under the Curve (AUC) metric across the ten folds tested.

The final performance of the selected model was assessed on the test subset of the data. Samples from the posterior distributions from the models for all ten folds were combined to predict the probability of HPAI infection. The probabilities were dichotomised using the mean optimal threshold value, again across all ten folds. Once the final predictions were done, the final AUC, sensitivity and specificity scores were calculated.

## Results

### Descriptive results

Monthly patterns in the primary and processed variables are visualised in Fig 2 and show HPAI outbreaks in 2016, 2017, 2018, and 2020–2022 (Fig 2A). The spatio-temporal rates aligned with this pattern (Fig 2C) but the means were low and variation was high. A generally high baseline mortality was seen in the wild bird mortality panels, Fig 2B, D. Peaks in mortality, most probably due to HPAI case fatalities were visible, but superimposed upon the baseline mortality. Notice also the low reported mortality in wild birds in 2017.

**Fig 3:**
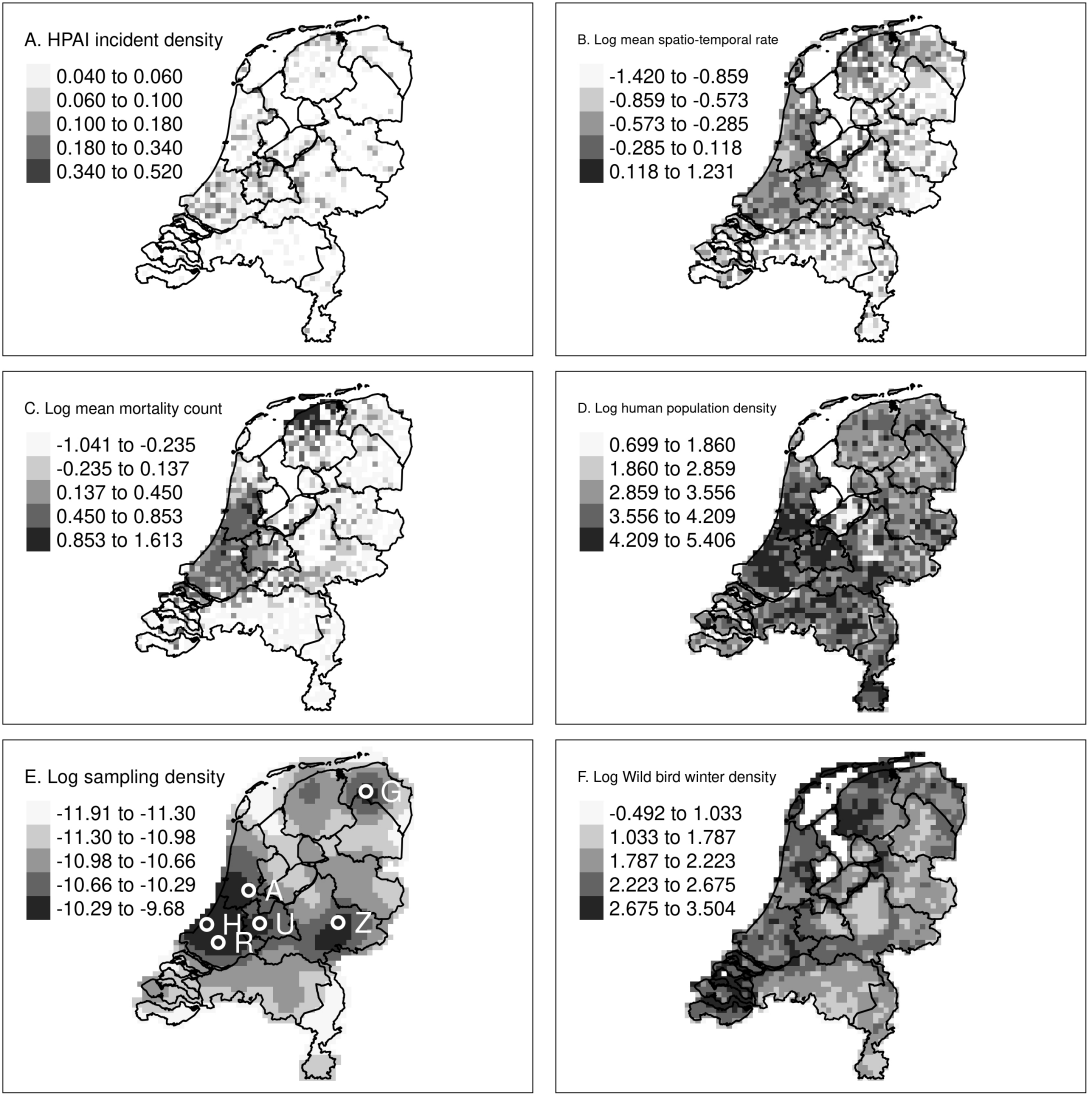
Spatial distribution of the variables. For temporal ranges, see Table 2. **A)** HPAI incident density, **B)** Mean log_10_ spatio-temporal rates for wild bird mortalities, **C)** Mean log_10_ wild bird mortality counts over 10 days within a 25 km radius, D) log_10_ human population density, E) log_10_ sampling density for wild bird mortalities, F) log_10_ summed wild bird density for 190 species of interest. Note the non-linear Fisher scaling of the legends. Abbreviations: A, Amsterdam; G, Groningen; H, Den Haag; R, Rotterdam; U, Utrecht; Z, Zutphen. *Boundary data: Statistics Netherlands* (*CBS*) and Kadaster Nederland; via the R package tmap.

Fig 3 shows the spatial distribution of the variables used in this analysis. Elevated HPAI incident densities were found in the west, middle, and north of the Netherlands, with spatio-temporal rates and wild bird mortality counts following this pattern. High human and sampling densities were in the west and middle but not in the north. High bird densities were present around the Wadden islands in the north and the delta in the south-west, and in northern mainland areas close to the coast.

The high sampling density west of the Amsterdam coincided with a high population density, but also to an area with an increased sampling effort by a single observer: Roy Slaterus, one of the authors. He intensely sampled the area west of Amsterdam, which we will refer to as “Roy’s hotspot”. It was consistently monitored, with Roy reporting bird mortalities monthly throughout the study period. Roy was responsible for 21% of the observations used in this analysis. Removing all Roy’s observations did not change the accuracy of our predictions as measured by the AUC (Table B in S1 Text). Collectively, the ten most active reporters contributed 39% of the observations, while 51% came from reporters who submitted only a single observation. The high sampling density around Rotterdam, Den Haag, Utrecht, Zutphen and Groningen, could not be attributed to a single observer.

**Fig 4:**
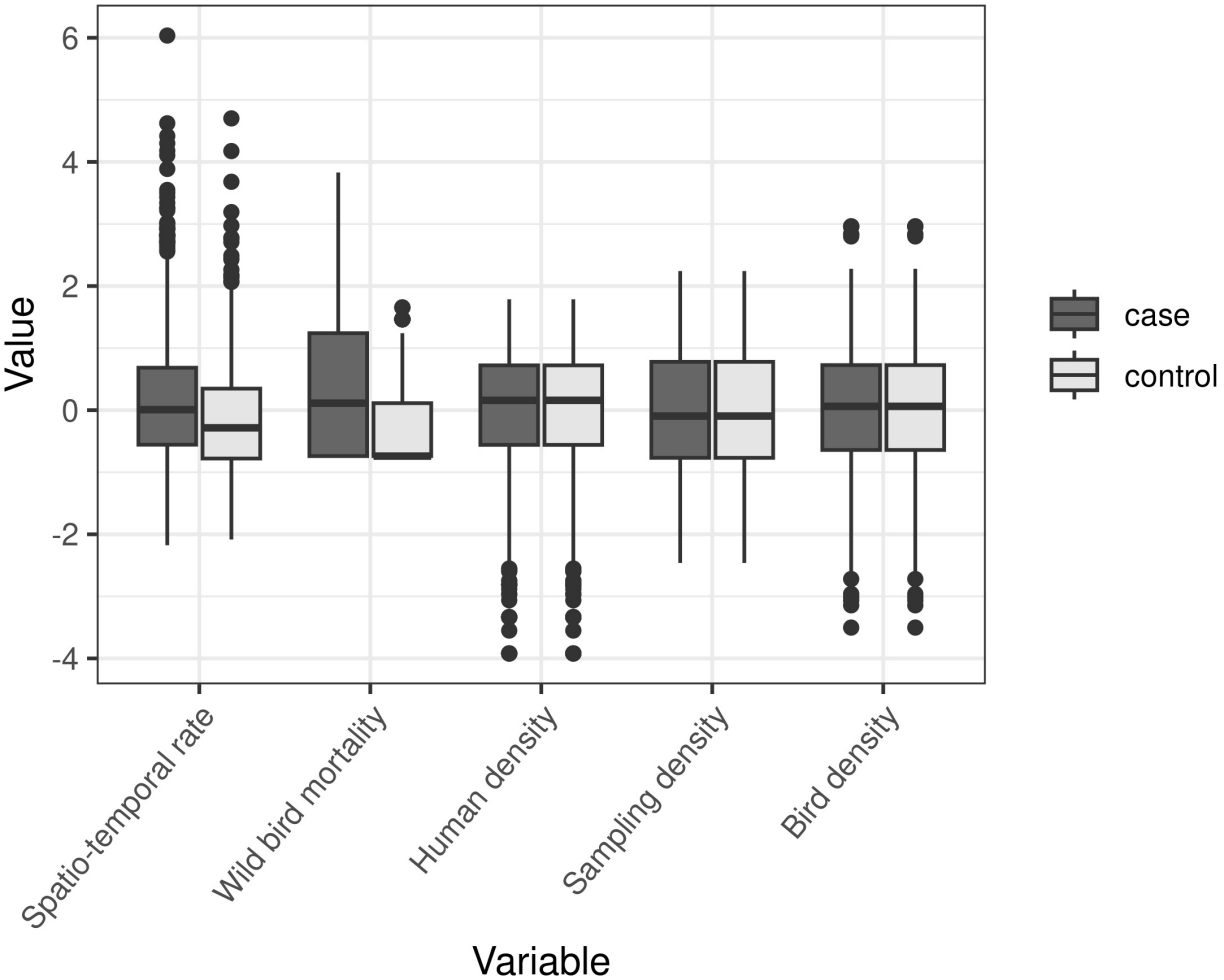
Standardized log_10_ values of the variables in the case-control dataset. Note that human, sampling, and bird densities are static variables and thus identical in both case and control situations. The spatio-temporal rate and wild bird mortality counts are elevated in the case situation.

Fig 4 summarises the values of the variables in the case-control dataset and shows an elevation of the median spatio-temporal rate and the median wild bird mortality count in the case situation. Human, sampling and bird densities are static variables and therefore are identical between the case and control situation.

### Model selection and performance

**Fig 5:**
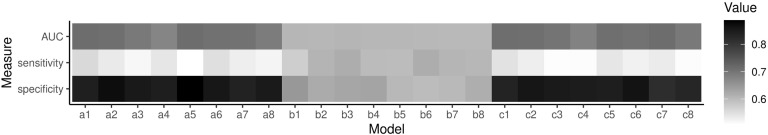
Mean performance measures from the ten-fold cross validation for all models. The highest AUC and specificity values were found in the a and c models. Sensitivity was higher in the b models. See Table 3 for model details.

Fig 5 shows a summary of the performance measures for all models (details in Figure A in S1 File and Table B in S1 File). The ten best performing models are presented in Table 4. Models based around spatial temporal distance (b1-b8) had the lowest AUC scores. In contrast, models based around spatio-temporal rates and mortality counts (a1-a8) or mortality counts alone (c1-c8) showed similar AUC performance.

**Table 4:** Ranking of the 10 best performing models by AUC score. The mean AUC is given with the range across the cross validation folds within parenthesis.

Rank	Model	AUC score
1	a1	0.71 (0.68 - 0.77)
2	a5	0.71 (0.68 - 0.77)
3	c7	0.71 (0.67 - 0.75)
4	c1	0.71 (0.68 - 0.76)
5	a2	0.71 (0.68 - 0.77)
6	c5	0.71 (0.68 - 0.76)
7	c2	0.71 (0.67 - 0.76)
8	a6	0.71 (0.67 - 0.76)
9	c6	0.7 (0.68 - 0.75)
10	a7	0.7 (0.66 - 0.75)

As can be seen in Table 4, the AUC performance of the top ten models was extremely similar. Further on in the manuscript, we will proceed using model c7, which is the simplest best performing model with only two variables: wild bird mortality (n) and bird density (B). Notice that all ten best performing models contained wild bird mortality (n), indicating the importance of this variable. Sampling density (D) was found in eight models, wild bird density (B) in six models, the spatio-temporal rate (ST) in five models and finally human density (H) was found in four models. The effect of mortality counts can also be seen in Fig 5, where the models in the ‘b’ family, all without mortality counts, had the lowest AUC scores.

**Fig 6:**
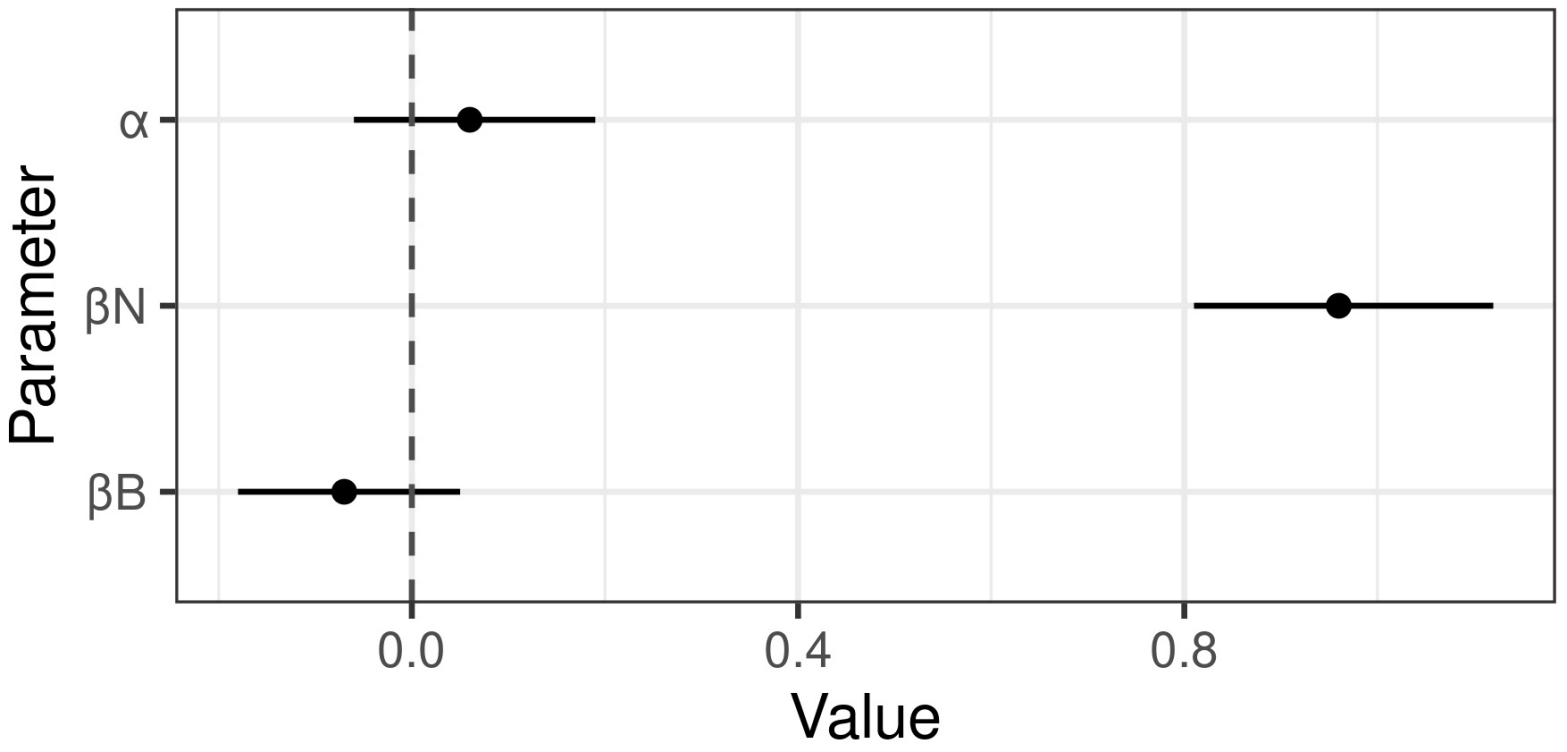
Posterior means and 95% compatibility intervals for the intercept (⍺) and slopes of the best performing model. The strongest effects is for the wild bird mortality counts (β*N).*

The final performance was assessed by making predictions on the test data. The mean threshold used for dichotomisation of probabilities produced by models c7 was 0.56. The resulting confusion matrix is provided as Table D in S1 File. The final sensitivity was 0.47 and the final specificity was 0.79. The strengths of the linear coefficients are available in Fig 6.

**Fig 7:**
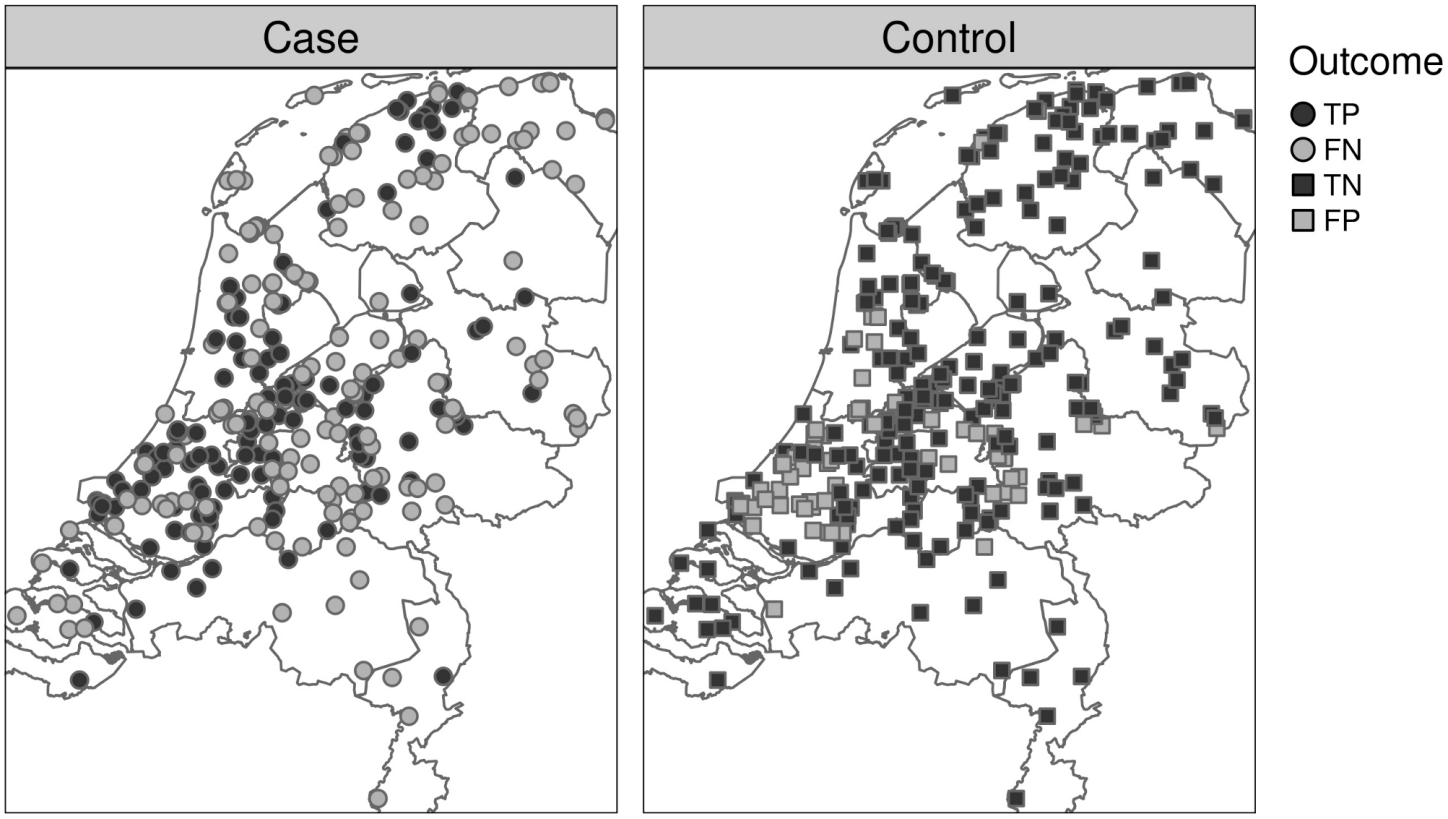
Spatial visualisation of model predictions for the test subset of the case-crossover data. As expected from the model sensitivity, cases are correctly marked as positive about half of the time. The majority of controls are marked correctly. Abbreviations: FN, false negative; FP, false positive; TN, true negative; TP, true positive. Boundary data: Statistics Netherlands (CBS) and Kadaster Nederland; via the R package tmap.

The spatial distribution of true positives, false negatives, true negatives and false negatives is shown in Fig 7. The effect of the chosen threshold on the sensitivity and specificity of the model is shown in Fig B in S1 Text. The relatively low sensitivity of the model is reflected in the presence of numerous false negatives. Model predictions for cases were correct in most areas of the Netherlands, except in the east of the Netherlands were relatively many false negatives were found. Model predictions for controls were mostly correct, except for the false positives found mostly in the highly populated areas in the west of the Netherlands.

### Spatio-temporal hotspots

**Fig 8:**
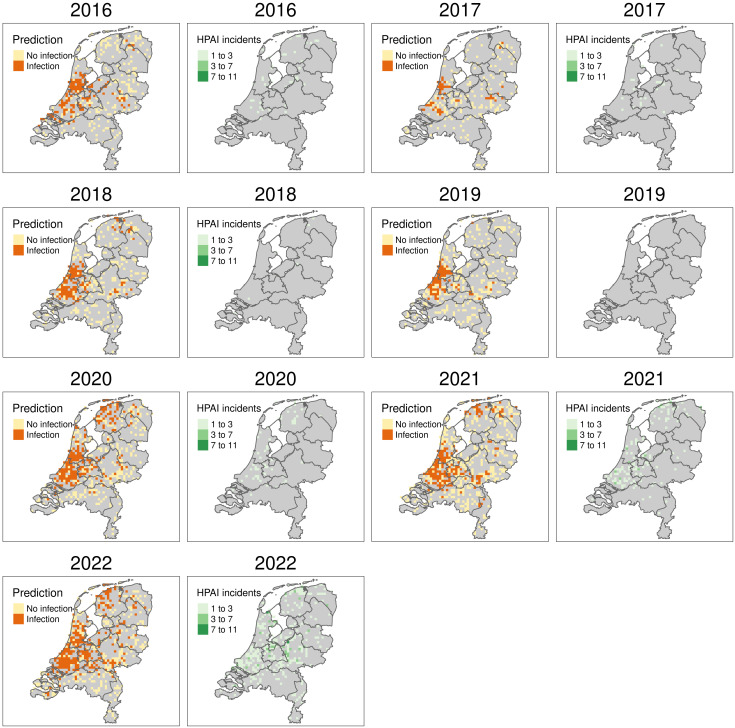
Yearly model predictions of HPAI in wild bird mortalities. For each year, a pair of figures is shown: model predictions on the left using a yellow to red colour gradient and HPAI incident counts on the right in green. Note that no HPAI incidents were reported in 2019. Result were aggregated using a 5x5 km grid. *Boundary data: Statistics Netherlands* (*CBS*) and Kadaster Nederland; via the R package tmap.

The best performing model, selected earlier, was used to make HPAI infection probability predictions on all wild bird mortalities reported (Fig 8). High infection probabilities were predicted west of Amsterdam from 2016 to 2019 in the area indicated as Roy’s hotspot before, implying that these predictions could be caused by Roy’s high sampling activity. From 2020 to 2022, outbreak risks expanded in the west and north, with higher risks in the north linked to increased mortality in ducks, swans, and geese. Notably, several HPAI cases were also detected in Roy’s hotspot during these later years.

**Fig 9:**
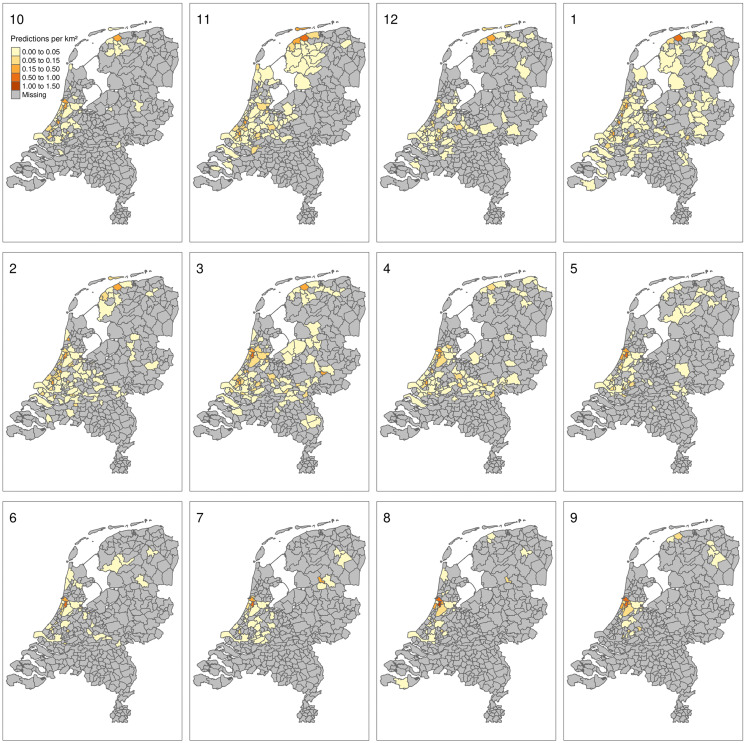
Monthly aggregated HPAI infection predictions for wild bird mortalities over the years 2016-2022. The number at the top of each panel indicates the month. Note that the series starts at the start of the HPAI season, October. Areas of higher risk are found from October to March and are low for the remainder of the year. Boundary data: Statistics Netherlands (CBS) and Kadaster Nederland; via the R package tmap.

We used the model to identify risk at municipal level on a monthly basis. The monthly predictions in Fig 9 begin in October, which marks the start of the epidemiological year for HPAI. The panels illustrate the spatial expansion of risks from November through April. From May onwards, less municipalities have predicted HPAI cases. As noted earlier, Roy’s hotspot is visible, especially during the months of May through October. There also appears to be a drop in reporting in December.

## Discussion

Our model effectively assigns HPAI infection probabilities to the locations of wild bird mortalities, demonstrating reasonable sensitivity and good specificity. The best-performing model, with the fewest variables, included wild bird mortality counts and wild bird density as variables. The yearly spatial distribution of predicted wild bird HPAI outbreak areas (Fig 8) generally matched lab-confirmed HPAI cases, particularly in 2021 and 2022. The highest number of monthly HPAI outbreak predictions in municipalities occurred from November through April (Fig 9).

Our results indicate that the model effectively identifies HPAI mortality hotspots in untested wild birds. This approach could offer valuable insights for risk managers, enabling them to spatially and temporally prioritize HPAI testing in both live and dead wild birds, as well as poultry farms. By focusing testing efforts on pre-selected areas and specific times of the year, the model could contribute to reduced sampling costs and the number of tests required. Additionally, targeted testing could increase species and geographical coverage. Our approach could also guide the implementation of preventive measures, such as housing orders, in high-risk areas and periods.

In this study, we haven’t focussed on individual bird species. Further investigation of the bird species within a mortality hotspot could provide important clues for future progression of the disease. A hotspot in mortality concerning mostly gregarious species could be the prelude for a much larger outbreak in that area and a hotspot with migratory species could imply a potential for a much larger spatial spread.

However, the model, as it has been trained now, cannot reliably be used as a tool for predicting HPAI-related wild bird mortality events in the future, due to the ever-changing nature of the virus. New strains may affect different bird species or cause varying levels of mortality compared to older strains [8,9]. Also, selective pressures exerted by HPAI outbreaks influence host susceptibility, further complicating predictions. The model’s primary value lies in retrospective identification of areas and seasons with a potential increase in the risk of HPAI virus circulation in wild birds. Since sampling live wild birds for viruses is labour-intensive and best conducted by specialists, we use mortality as an indicator of HPAI circulation. By understanding the areas and conditions that facilitate HPAI spread, measured by mortality, we can look for patterns in wild bird ecology and HPAI virus behaviour [36]. This knowledge can then be used to inform future outbreak predictions.

The model relies on passive surveillance data from wild bird mortality reports and wild birds tested for HPAI. The strength of this approach is the large number of observers and observations. Its weakness, however, lies in the spatially and temporally unstructured nature of the sampling. As a result, highly populated areas in the west of the Netherlands are sampled more intensively than the less populated east (Fig 3E). Additionally, a single very active observer can cause the model to flag areas as risky, as seen by Roy’s hotspot (Fig 8). Finally, reporting can be influenced by societal factors, like the timing of the holidays (Fig 9).

Both human and wild bird densities were included in the models as static variables. This is appropriate for the medium-short time range of this study. For longer duration studies, it would be beneficial to regularly update these data.

By including the spatio-temporal rate, we aimed to make the model specific to HPAI outbreaks. However, the importance of this factor among the ten best-performing models was low compared to the importance of wild bird mortality counts, a very generic variable. As a result, the model may generate false positives whenever many dead birds are reported due to other causes, such as increased volunteer activity, cold weather-induced mortality, other diseases like botulism, or food shortages. This property of the model could be improved when the model is updated in the years to come. When wild bird mortality occurs for other reasons than HPAI, the spatio-temporal distance measure will gain more weight in the model and predictions can potentially become more HPAI specific.

Our results are specific to the Dutch situation and the model cannot be used as-is in other countries. Our final model contained the variables for wild bird density and mortality counts. In another country this could turn out to be differently. In addition, this could even change in the Dutch situation as data is added over time, as mentioned in the previous paragraph. The analysis framework, however, can be applied to any other country or region that has passive surveillance of wild bird mortality and testing for HPAI cases. The challenges of using volunteer data remain the same, but so do the opportunities.

For future research, it would be interesting to explore the effectiveness and costs of the current wild bird reporting and testing scheme with a scheme where hotspot areas are tested as indicated by our model. In addition, it would also be interesting to further investigate combining passive with active surveillance in the hotspots identified using this model.

## Conclusions

Our study demonstrates that leveraging citizen science data on wild bird mortality enables effective identification of high-risk areas for HPAI outbreaks. This approach can support timely outbreak response strategies and targeted testing, potentially reducing costs and enhancing surveillance efficiency. While specific to the Dutch context, our analysis framework offers a valuable model adaptable to other regions with similar surveillance capabilities.

## Supporting information

S1 File Supplementary tables and figures. (DOCX)

S2 Text Wild bird density modelling methodology. (DOCX)
