# Supplementary material for: Identifying avian influenza hotspots in wild birds in the Netherlands
Source: PLoS One. 2026 Feb 12;21(2):e0341829. doi: 10.1371/journal.pone.0341829 (PMC12900324; doi:10.1371/journal.pone.0341829)
Supplement: S1 File — (DOCX) [file pone.0341829.s001.docx]

Identifying avian influenza hotspots in wild birds in the Netherlands

S1 File

Ronald Petie, Eduardo de Freitas Costa, Christian Kampichler, Roy Slaterus and Jose L. Gonzales

## Table A

Included bird species.

| EURING | Species | Scientific name | Group |
| --- | --- | --- | --- |
| 20 | Red-throated diver | *Gavia stellata* | Sea and coastal birds |
| 30 | Black-throated diver | *Gavia arctica* | Sea and coastal birds |
| 40 | Great northern diver | *Gavia immer* | Sea and coastal birds |
| 70 | Little grebe | *Tachybaptus ruficollis* | Grebes |
| 90 | Great crested grebe | *Podiceps cristatus* | Grebes |
| 100 | Red-necked grebe | *Podiceps grisegena* | Grebes |
| 110 | Horned grebe | *Podiceps auritus* | Grebes |
| 120 | Black-necked grebe | *Podiceps nigricollis* | Grebes |
| 220 | Northern fulmar | *Fulmarus glacialis* | Sea and coastal birds |
| 430 | Sooty shearwater | *Ardenna grisea* | Sea and coastal birds |
| 460 | Manx shearwater | *Puffinus puffinus sensu lato* | Sea and coastal birds |
| 461 | Manx shearwater | *Puffinus puffinus* | Sea and coastal birds |
| 520 | European storm petrel | *Hydrobates pelagicus* | Sea and coastal birds |
| 550 | Leach's storm petrel | *Hydrobates leucorhous* | Sea and coastal birds |
| 710 | Northern gannet | *Morus bassanus* | Sea and coastal birds |
| 720 | Great cormorant | *Phalacrocorax carbo* | Other |
| 800 | European shag | *Gulosus aristotelis* | Sea and coastal birds |
| 950 | Eurasian bittern | *Botaurus stellaris* | Other |
| 980 | Little bittern | *Ixobrychus minutus* | Other |
| 1040 | Black-crowned night heron | *Nycticorax nycticorax* | Other |
| 1110 | Cattle egret | *Bubulcus ibis* | Other |
| 1190 | Little egret | *Egretta garzetta* | Other |
| 1210 | Great egret | *Ardea alba* | Other |
| 1220 | Grey heron | *Ardea cinerea* | Other |
| 1240 | Purple heron | *Ardea purpurea* | Other |
| 1310 | Black stork | *Ciconia nigra* | Other |
| 1340 | White stork | *Ciconia ciconia* | Other |
| 1360 | Glossy ibis | *Plegadis falcinellus* | Other |
| 1420 | Sacred ibis | *Threskiornis aethiopicus* | Other |
| 1440 | Eurasian spoonbill | *Platalea leucorodia* | Other |
| 1470 | Greater flamingo | *Phoenicopterus ruber sensu lato* | Other |
| 1478 | Chilean flamingo | *Phoenicopterus chilensis* | Other |
| 1520 | Mute swan | *Cygnus olor* | Ducks, geese and swans |
| 1528 | Black swan | *Cygnus atratus* | Ducks, geese and swans |
| 1530 | Tundra swan | *Cygnus columbianus* | Ducks, geese and swans |
| 1540 | Whooper swan | *Cygnus cygnus* | Ducks, geese and swans |
| 1560 | Swan goose | *Anser cygnoides* | Ducks, geese and swans |
| 1571 | Taiga bean goose | *Anser fabalis* | Ducks, geese and swans |
| 1574 | Tundra bean goose | *Anser serrirostris* | Ducks, geese and swans |
| 1580 | Pink-footed goose | *Anser brachyrhynchus* | Ducks, geese and swans |
| 1590 | Greater white-fronted goose | *Anser albifrons* | Ducks, geese and swans |
| 1600 | Lesser white-fronted goose | *Anser erythropus* | Ducks, geese and swans |
| 1610 | Greylag goose | *Anser anser* | Ducks, geese and swans |
| 1620 | Bar-headed goose | *Anser indicus* | Ducks, geese and swans |
| 1630 | Snow goose | *Anser caerulescens* | Ducks, geese and swans |
| 1650 | Emperor goose | *Anser canagicus* | Ducks, geese and swans |
| 1661 | Canada goose | *Branta canadensis canadensis* | Ducks, geese and swans |
| 1664 | Cackling goose | *Branta hutchinsii* | Ducks, geese and swans |
| 1670 | Barnacle goose | *Branta leucopsis* | Ducks, geese and swans |
| 1680 | Brent goose | *Branta bernicla* | Ducks, geese and swans |
| 1682 | Pale-bellied brent goose | *Branta bernicla hrota* | Ducks, geese and swans |
| 1683 | Black brant | *Branta bernicla nigricans* | Ducks, geese and swans |
| 1690 | Red-breasted goose | *Branta ruficollis* | Ducks, geese and swans |
| 1700 | Egyptian goose | *Alopochen aegyptiaca* | Ducks, geese and swans |
| 1710 | Ruddy shelduck | *Tadorna ferruginea* | Ducks, geese and swans |
| 1730 | Common shelduck | *Tadorna tadorna* | Ducks, geese and swans |
| 1750 | Muscovy duck | *Cairina moschata* | Ducks, geese and swans |
| 1770 | Wood duck | *Aix sponsa* | Ducks, geese and swans |
| 1780 | Mandarin duck | *Aix galericulata* | Ducks, geese and swans |
| 1790 | Eurasian wigeon | *Mareca penelope* | Ducks, geese and swans |
| 1820 | Gadwall | *Mareca strepera* | Ducks, geese and swans |
| 1840 | Eurasian teal | *Anas crecca* | Ducks, geese and swans |
| 1860 | Mallard | *Anas platyrhynchos* | Ducks, geese and swans |
| 1890 | Northern pintail | *Anas acuta* | Ducks, geese and swans |
| 1910 | Garganey | *Spatula querquedula* | Ducks, geese and swans |
| 1940 | Northern shoveler | *Spatula clypeata* | Ducks, geese and swans |
| 1960 | Red-crested pochard | *Netta rufina* | Ducks, geese and swans |
| 1980 | Common pochard | *Aythya ferina* | Ducks, geese and swans |
| 2020 | Ferruginous duck | *Aythya nyroca* | Ducks, geese and swans |
| 2030 | Tufted duck | *Aythya fuligula* | Ducks, geese and swans |
| 2040 | Greater scaup | *Aythya marila* | Ducks, geese and swans |
| 2060 | Common eider | *Somateria mollissima* | Sea and coastal birds |
| 2120 | Long-tailed duck | *Clangula hyemalis* | Sea and coastal birds |
| 2130 | Common scoter | *Melanitta nigra* | Sea and coastal birds |
| 2150 | Velvet scoter | *Melanitta fusca* | Sea and coastal birds |
| 2180 | Common goldeneye | *Bucephala clangula* | Ducks, geese and swans |
| 2200 | Smew | *Mergellus albellus* | Ducks, geese and swans |
| 2210 | Red-breasted merganser | *Mergus serrator* | Ducks, geese and swans |
| 2230 | Goosander | *Mergus merganser* | Ducks, geese and swans |
| 2250 | Ruddy duck | *Oxyura jamaicensis* | Ducks, geese and swans |
| 2310 | European honey buzzard | *Pernis apivorus* | Raptors and owls |
| 2380 | Black kite | *Milvus migrans* | Raptors and owls |
| 2390 | Red kite | *Milvus milvus* | Raptors and owls |
| 2430 | White-tailed eagle | *Haliaeetus albicilla* | Raptors and owls |
| 2510 | Griffon vulture | *Gyps fulvus* | Raptors and owls |
| 2560 | Short-toed snake eagle | *Circaetus gallicus* | Raptors and owls |
| 2600 | Western marsh harrier | *Circus aeruginosus* | Raptors and owls |
| 2610 | Hen harrier | *Circus cyaneus* | Raptors and owls |
| 2620 | Pallid harrier | *Circus macrourus* | Raptors and owls |
| 2630 | Montagu's harrier | *Circus pygargus* | Raptors and owls |
| 2670 | Northern goshawk | *Accipiter gentilis* | Raptors and owls |
| 2690 | Eurasian sparrowhawk | *Accipiter nisus* | Raptors and owls |
| 2870 | Common buzzard | *Buteo buteo* | Raptors and owls |
| 2900 | Rough-legged buzzard | *Buteo lagopus* | Raptors and owls |
| 3010 | Osprey | *Pandion haliaetus* | Raptors and owls |
| 3040 | Common kestrel | *Falco tinnunculus* | Raptors and owls |
| 3070 | Red-footed falcon | *Falco vespertinus* | Raptors and owls |
| 3090 | Merlin | *Falco columbarius* | Raptors and owls |
| 3100 | Eurasian hobby | *Falco subbuteo* | Raptors and owls |
| 3200 | Peregrine falcon | *Falco peregrinus* | Raptors and owls |
| 4070 | Water rail | *Rallus aquaticus* | Other |
| 4080 | Spotted crake | *Porzana porzana* | Other |
| 4100 | Little crake | *Zapornia parva* | Other |
| 4110 | Baillon's crake | *Zapornia pusilla* | Other |
| 4210 | Corn crake | *Crex crex* | Other |
| 4240 | Common moorhen | *Gallinula chloropus* | Other |
| 4290 | Eurasian coot | *Fulica atra* | Other |
| 4330 | Common crane | *Grus grus* | Other |
| 4500 | Eurasian oystercatcher | *Haematopus ostralegus* | Waders |
| 4550 | Black-winged stilt | *Himantopus himantopus* | Waders |
| 4560 | Pied avocet | *Recurvirostra avosetta* | Waders |
| 4590 | Eurasian stone-curlew | *Burhinus oedicnemus* | Waders |
| 4690 | Little ringed plover | *Charadrius dubius* | Waders |
| 4700 | Common ringed plover | *Charadrius hiaticula* | Waders |
| 4770 | Kentish plover | *Charadrius alexandrinus* | Waders |
| 4820 | Eurasian dotterel | *Charadrius morinellus* | Waders |
| 4850 | European golden plover | *Pluvialis apricaria* | Waders |
| 4860 | Grey plover | *Pluvialis squatarola* | Waders |
| 4930 | Northern lapwing | *Vanellus vanellus* | Waders |
| 4960 | Red knot | *Calidris canutus* | Waders |
| 4970 | Sanderling | *Calidris alba* | Waders |
| 5010 | Little stint | *Calidris minuta* | Waders |
| 5020 | Temminck's stint | *Calidris temminckii* | Waders |
| 5070 | Pectoral sandpiper | *Calidris melanotos* | Waders |
| 5090 | Curlew sandpiper | *Calidris ferruginea* | Waders |
| 5100 | Purple sandpiper | *Calidris maritima* | Waders |
| 5120 | Dunlin | *Calidris alpina* | Waders |
| 5140 | Broad-billed sandpiper | *Calidris falcinellus* | Waders |
| 5170 | Ruff | *Calidris pugnax* | Waders |
| 5180 | Jack snipe | *Lymnocryptes minimus* | Waders |
| 5190 | Common snipe | *Gallinago gallinago* | Waders |
| 5290 | Eurasian woodcock | *Scolopax rusticola* | Waders |
| 5320 | Black-tailed godwit | *Limosa limosa* | Waders |
| 5340 | Bar-tailed godwit | *Limosa lapponica* | Waders |
| 5380 | Whimbrel | *Numenius phaeopus* | Waders |
| 5410 | Eurasian curlew | *Numenius arquata* | Waders |
| 5450 | Spotted redshank | *Tringa erythropus* | Waders |
| 5460 | Common redshank | *Tringa totanus* | Waders |
| 5470 | Marsh sandpiper | *Tringa stagnatilis* | Waders |
| 5480 | Common greenshank | *Tringa nebularia* | Waders |
| 5530 | Green sandpiper | *Tringa ochropus* | Waders |
| 5540 | Wood sandpiper | *Tringa glareola* | Waders |
| 5560 | Common sandpiper | *Actitis hypoleucos* | Waders |
| 5610 | Ruddy turnstone | *Arenaria interpres* | Waders |
| 5640 | Red-necked phalarope | *Phalaropus lobatus* | Waders |
| 5650 | Grey phalarope | *Phalaropus fulicarius* | Waders |
| 5660 | Pomarine jaeger | *Stercorarius pomarinus* | Sea and coastal birds |
| 5670 | Parasitic jaeger | *Stercorarius parasiticus* | Sea and coastal birds |
| 5680 | Long-tailed jaeger | *Stercorarius longicaudus* | Sea and coastal birds |
| 5690 | Great skua | *Stercorarius skua* | Sea and coastal birds |
| 5750 | Mediterranean gull | *Ichthyaetus melanocephalus* | Gulls and terns |
| 5780 | Little gull | *Hydrocoloeus minutus* | Gulls and terns |
| 5790 | Sabine's gull | *Xema sabini* | Gulls and terns |
| 5820 | Black-headed gull | *Chroicocephalus ridibundus* | Gulls and terns |
| 5900 | Mew gull | *Larus canus* | Gulls and terns |
| 5910 | Lesser black-backed gull | *Larus fuscus* | Gulls and terns |
| 5920 | Herring gull | *Larus argentatus agg.* | Gulls and terns |
| 5925 | Yellow-legged gull | *Larus michahellis atlantis* | Gulls and terns |
| 5926 | Yellow-legged gull | *Larus michahellis* | Gulls and terns |
| 5980 | Iceland gull | *Larus glaucoides* | Gulls and terns |
| 5990 | Glaucous gull | *Larus hyperboreus* | Gulls and terns |
| 6000 | Great black-backed gull | *Larus marinus* | Gulls and terns |
| 6020 | Black-legged kittiwake | *Rissa tridactyla* | Gulls and terns |
| 6050 | Gull-billed tern | *Gelochelidon nilotica* | Gulls and terns |
| 6060 | Caspian tern | *Hydroprogne caspia* | Gulls and terns |
| 6110 | Sandwich tern | *Thalasseus sandvicensis* | Gulls and terns |
| 6150 | Common tern | *Sterna hirundo* | Gulls and terns |
| 6160 | Arctic tern | *Sterna paradisaea* | Gulls and terns |
| 6240 | Little tern | *Sternula albifrons* | Gulls and terns |
| 6260 | Whiskered tern | *Chlidonias hybrida* | Gulls and terns |
| 6279 | Black tern | *Chlidonias niger* | Gulls and terns |
| 6280 | White-winged tern | *Chlidonias leucopterus* | Gulls and terns |
| 6340 | Common murre | *Uria aalge* | Sea and coastal birds |
| 6360 | Razorbill | *Alca torda* | Sea and coastal birds |
| 6380 | Black guillemot | *Cepphus grylle* | Sea and coastal birds |
| 6470 | Little auk | *Alle alle* | Sea and coastal birds |
| 6540 | Atlantic puffin | *Fratercula arctica* | Sea and coastal birds |
| 7350 | Barn owl | *Tyto alba* | Raptors and owls |
| 7440 | Eurasian eagle-owl | *Bubo bubo* | Raptors and owls |
| 7570 | Little owl | *Athene noctua* | Raptors and owls |
| 7610 | Tawny owl | *Strix aluco* | Raptors and owls |
| 7670 | Long-eared owl | *Asio otus* | Raptors and owls |
| 7680 | Short-eared owl | *Asio flammeus* | Raptors and owls |
| 15390 | Eurasian jay | *Garrulus glandarius* | Crows |
| 15490 | Eurasian magpie | *Pica pica* | Crows |
| 15600 | Western jackdaw | *Coloeus monedula* | Crows |
| 15630 | Rook | *Corvus frugilegus* | Crows |
| 15671 | Carrion crow | *Corvus corone* | Crows |
| 15673 | Hooded crow | *Corvus cornix* | Crows |
| 15720 | Common raven | *Corvus corax* | Crows |

**Description:** For the analysis only bird species susceptible to Highly Pathogenic Avian Influenza (HPAI) were included. For each species the EURING bird species code ([https://euring.org/](#X6589fc6ab0dc82cf12099d1c2d40ab994e8410c)) is given, as well as the English and scientific species name and a grouping.

## Table B

Effect of time window and reporting on AUC.

| Description | AUC | Z | p-value |
| --- | --- | --- | --- |
| Standard analysis | 0.68 (0.64-0.72) | 0.000 | 1.000 |
| Exclude Roy's observations | 0.69 (0.64-0.73) | -0.922 | 0.356 |
| 41 day time window | 0.7 (0.66-0.74) | -1.910 | 0.056 |
| 61 day time window | 0.71 (0.67-0.76) | -2.278 | 0.023 |
| 81 day time window | 0.71 (0.67-0.75) | -1.691 | 0.091 |

**Description:** Comparison of AUC scores. The standard analysis with a 21 day time window for counting bird mortalities was compared to four other scenario’s. First, excluding the wild bird mortalities reported by Roy Slaterus. In scenario three until five, only the duration of the time window was increased. AUC scores are given with a bootstrapped (n = 1000) 2.5-97.5% confidence interval between parenthesis. Z scores and p-values are given for the comparisons of the Receiver Operator Curves (ROC) with the standard analysis, using the ‘delong’ method. Taking into account the Bonferroni correction, the significance threshold for this five-way comparison is 0.05/5 = 0.01. This means that none of the scenario’s are significantly different from the standard analysis.

## Figure A

| 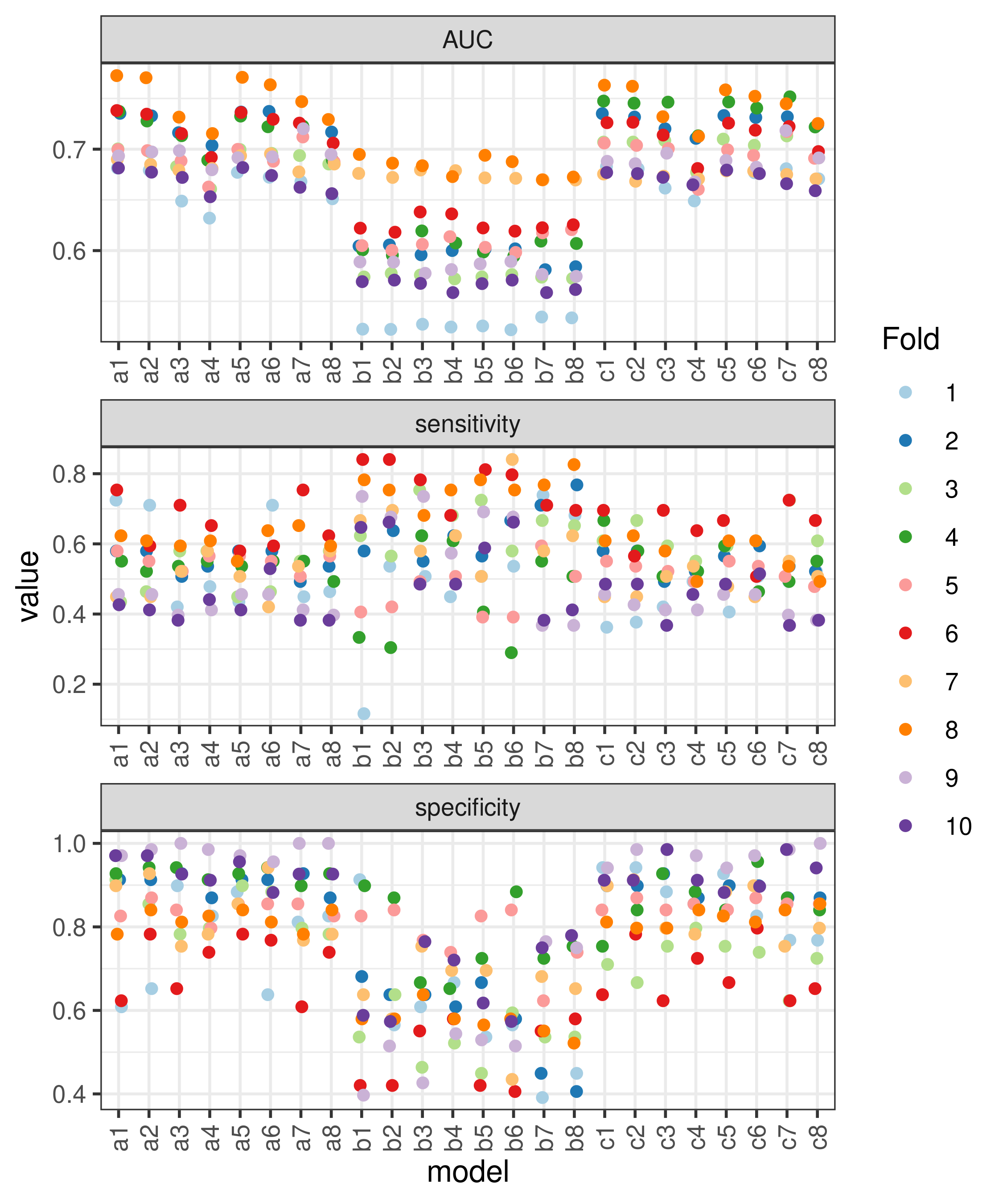  Supplementary figure 1. Model comparison. Area under the curve (top), sensitivity (middle) and specificity (bottom). Models a1-a8 contain both the spatio-temporal rate (ST) and wild bird mortality count (n), models b1-b8 contain only ST and models c1-c8 contain only n. The index of the ten fold cross validation fold is given by the colour. See Table 3 in the main manuscript for more details on included variables. |
| --- |

## Table C

Detailed rankings based on AUC.

| Rank | Model | Min | Mean | Max |
| --- | --- | --- | --- | --- |
| 1 | a1 | 0.681 | 0.713 | 0.773 |
| 2 | a5 | 0.677 | 0.712 | 0.771 |
| 3 | c7 | 0.666 | 0.712 | 0.752 |
| 4 | c1 | 0.675 | 0.711 | 0.763 |
| 5 | a2 | 0.677 | 0.710 | 0.770 |
| 6 | c5 | 0.679 | 0.710 | 0.758 |
| 7 | c2 | 0.668 | 0.709 | 0.762 |
| 8 | a6 | 0.672 | 0.707 | 0.763 |
| 9 | c6 | 0.676 | 0.705 | 0.752 |
| 10 | a7 | 0.662 | 0.705 | 0.747 |
| 11 | c3 | 0.662 | 0.702 | 0.746 |
| 12 | a3 | 0.649 | 0.695 | 0.732 |
| 13 | c8 | 0.659 | 0.694 | 0.725 |
| 14 | a8 | 0.651 | 0.690 | 0.729 |
| 15 | c4 | 0.649 | 0.681 | 0.713 |
| 16 | a4 | 0.632 | 0.677 | 0.715 |
| 17 | b3 | 0.527 | 0.607 | 0.684 |
| 18 | b1 | 0.523 | 0.606 | 0.695 |
| 19 | b4 | 0.525 | 0.605 | 0.679 |
| 20 | b5 | 0.526 | 0.605 | 0.694 |
| 21 | b2 | 0.522 | 0.604 | 0.686 |
| 22 | b6 | 0.522 | 0.603 | 0.688 |
| 23 | b8 | 0.534 | 0.602 | 0.673 |
| 24 | b7 | 0.535 | 0.601 | 0.670 |

**Description:** Area under the Curve (AUC) score summary. For each model ten model iterations were trained, one on each of the folds in the ten fold cross validation. Here, the summary statistics are given.

## Figure B

The effect of the chosen threshold on the sensitivity and specificity of the model.

| 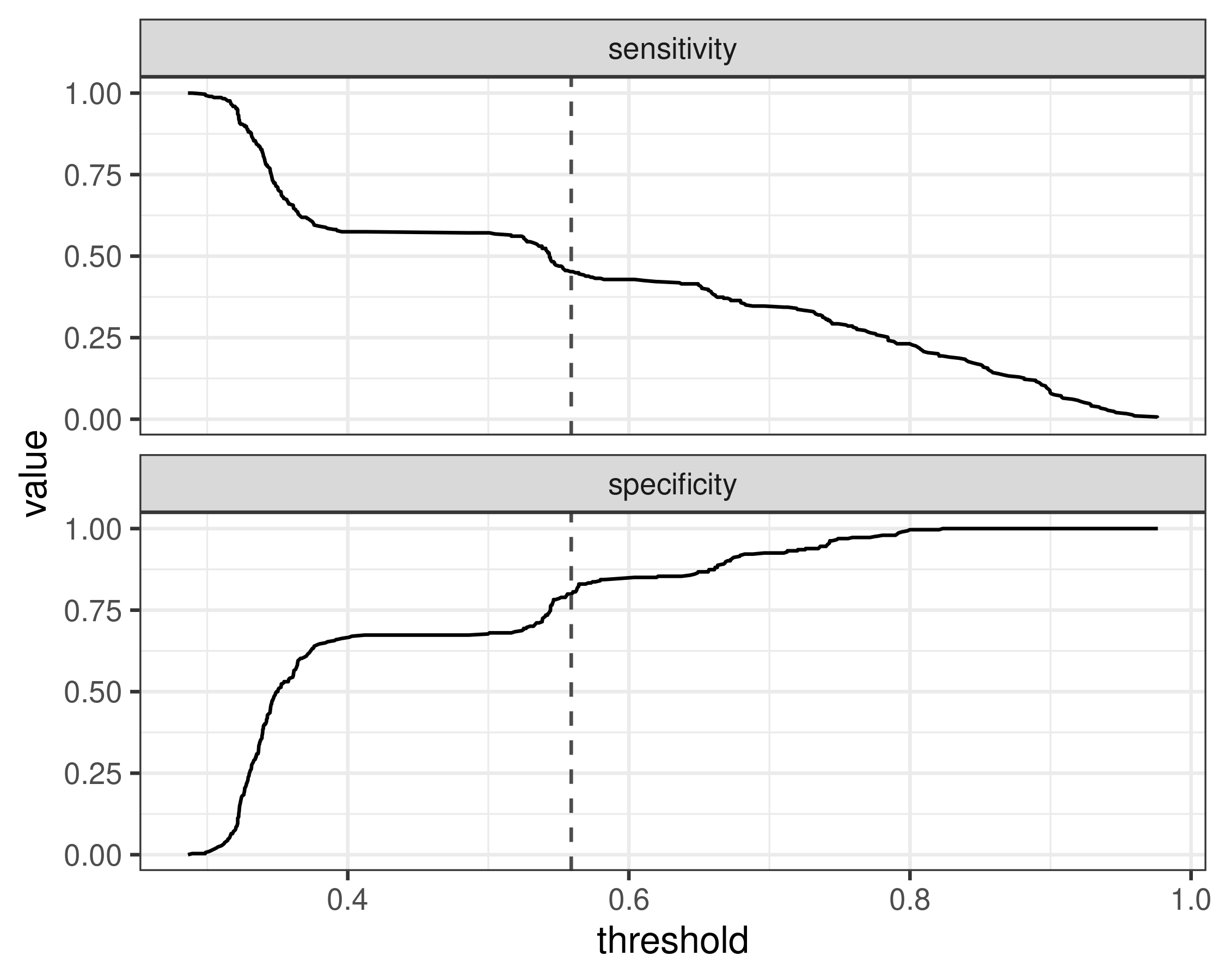  Supplementary figure 2. The sensitivity (top panel) and specificity (bottom panel) as a function of the threshold used the dichotomise the model outcomes. |
| --- |

**Description:** The behaviour of the sensitivity and specificity as a function of the dichotomisation threshold is highly non-linear. The optimal threshold was determined by the cutpointr package using the standard ‘maximize metric’ option and was found at the end of plateau, before further decreases in sensitivity and increases in specificity.

## Table D

Confusion matrix for final performance assessment. Abbreviations: TN, true negative; FP, false positive; FN, false negative; TP, true positive.

| Truth | Prediction | n | Label |
| --- | --- | --- | --- |
| FALSE | FALSE | 231 | TN |
| FALSE | TRUE | 63 | FP |
| TRUE | FALSE | 156 | FN |
| TRUE | TRUE | 138 | TP |

**Description:** The best performing model and the optimal threshold were used to predict HPAI infectious status in the test subset of the case-crossover data. Sensitivity and specificity values are given in the main paper. The confusion matrix is presented here.
