# Supplementary material for: Identifying avian influenza hotspots in wild birds in the Netherlands
Source: PLoS One. 2026 Feb 12;21(2):e0341829. doi: 10.1371/journal.pone.0341829 (PMC12900324; doi:10.1371/journal.pone.0341829)
Supplement: S2 Text — (DOCX) [file pone.0341829.s002.docx]

Identifying avian influenza hotspots in wild birds in the Netherlands

Supplementary information on wild bird abundance modelling

Ronald Petie, Eduardo de Freitas Costa, Christian Kampichler, Roy Slaterus and Jose L. Gonzales

## Data sources

Bird data for abundance modeling were mainly collected in the course of various monitoring schemes that are operated by the Sovon Dutch Centre for Field Ornithologie ([https://www.sovon.nl](https://www.sovon.nl/)). Each scheme has its own counting protocol and focuses on a unique aspect of the Dutch avifauna. Below is a brief summary of the various projects from which data originates:

*Waterfowl Monitoring Network* (Hornman et al. 2023)

The national waterfowl counts are part of the Ecological Monitoring Network (NEM), the system of nature monitoring networks of the government (Centraal Bureau voor de Statistiek 2012). The monitoring network is a partnership between Sovon, the National Water Agency Rijkswaterstaat (<https://www.rijkswaterstaat.nl/en>), the former Dutch Ministry of Economic Affairs, Agriculture and Innovation (now Ministry of Agriculture, Fishing, Food Security and Nature, <https://www.rijksoverheid.nl/ministeries/ministerie-van-landbouw-visserij-voedselzekerheid-en-natuur>) and the Dutch Central Bureau of Statistics (CBS, <https://www.cbs.nl/en-gb>). It consists of two major sub schemes:

- Monthly counts in monitoring areas
  In a sample of monitoring areas, including almost all larger (inter)nationally important water bodies, all waterfowl species are counted. Besides, geese and swans are counted in important foraging areas ('goose areas'), often in agricultural areas. The vast majority of these areas are counted monthly from September to April, the Wadden Sea even all year round.
- Midwinter count in mid-January
  During this count as many areas as possible are investigated complementing the monitoring areas, as well as concentrations of scoters in the Wadden Sea and the North Sea.

The counts take place in the weekend around the middle of the month, in tidal areas sometimes a weekend earlier or later due to the tide. The majority of counts is carried out by volunteers. Professionals make an important contribution in areas covered by volunteers cannot be properly examined (including aircraft counts of Eider and scoters in the Wadden Sea). Fixed counting areas are used that are visited during the day or around the time of high water (tidal areas) and must be counted in its entirety.

It is particularly relevant that the count data were also used to give an indication of the population size of waterfowl occurring in the Netherlands (Hornman et al. 2012, 2015). For this purpose, the counted and estimated numbers in the monitoring areas, the counted and estimated numbers in the other count areas (including those counted during the midwinter count) and an estimate of the numbers in areas that were not counted were added up per month and per year.

*Transect-Point Counts* (van Manen & de Jong, 2024)

This monitoring schemes consists of fixed routes that prefereably have to be visited in December but may also be visited in January. A route consists of twenty counting points that are at least 250 metres apart. At each counting point, all birds have to be recorded for exactly five minutes. Birds that are observed outside the five-minute period are not counted.

*Bird Atlas of the Netherlands* (Sovon 2018)

Data were used from the most recent 'Bird Atlas of the Netherlands'. For this atlas winter fieldwork took place in the winters from 2012/2013 – 2014/2015 and partly in 2015/2016. As with previous atlases, the work took place in 5 x 5 km atlas blocks within which a so-called 'golden grid' of eight fixed kilometre squares were counted. Counters determine for each kilometre square a complete list of species present and provided estimates of the number of individuals per species.

*LiveAtlas* (de Jong et al., 2023)

LiveAtlas is Sovon's latest project, and in some ways sentence the sequel to the last Bird Atlas of the Netherlands (Sovon 2018). It is a project in which all types of birds are counted in all seasons and has been running since 2018. It does not use fixed observation areas such as in the Sovon Breeding Monitoring Project or a ‘Golden grid’ such as in the fieldwork for the Bird Atlas of the Netherlands. Instead, volunteers are allowed to freely choose kilometre squares, whereby in theory the aim is to achieve national coverage. Within a kilometre square a route is recorded (‘track’) with the individual observations of species linked to it, all of which are provided with their own coordinate data. The data from Live Atlas comprise count data but also presence/absence data.

Because LiveAtlas is a year-round project, it contains a lot of valuable information on the occurrence and numbers of birds in months that are less well received other counting projects; this is also the case for winter Birds.

*Roosting Monitoring Network* (Hornman et al., 2023)

For some species the data were completed by records made in the course of the Roosting Monitoring Network because these species have better coverage when counted on sleeping places than when they forage in less covered agricultural areas.

## Environmental variables and modelling

In the regression between observations and environment, 112 variables were used. They consisted of climate variables (Bioclim dataset), water-related variables, habitat types, land use variables, crop types and infrastructure from a variety of data sources on a grid of 250 x 250 m. The spatial model is constructed by a combination of a regression model and the spatially interpolated residuals of the regression model, a method called *regression-kriging* (Hengl et al., 2009; Sierdsema & van Loon, 2008) for more information about this methodology. Random forest models were used for the spatial analysis of the breeding birds (Breiman 2001, Boulesteix et al. 2012).

Random forests are suitable for this analysis because they can handle high-dimensional, nonlinear and collinear data and because they are less susceptible to overfitting than other modelling techniques. Random forests are based on the idea of ​​training a large number of regression trees. Regression trees are a classic machine learning method that was developed four decades ago (Breiman et al. 1984). For each of the *n* trees in a random forest, only a bootstrapped sample of the observations is used and in each bifurcation of the tree only a randomly chosen subset of the explanatory variables is chosen. Each tree in the random forest will therefore yield different predictions, depending on the cases and environmental variables used. Finally, the average prediction of the *n* trees is calculated for each observation. Random forests are frequently used for species distribution modeling and similar analyses (e.g., Benito Garzon et al. 2006, Kampichler et al. 2010, Mascaro et al. 2014, Bellin et al. 2022, Ebrahimi et al. 2023) and in recent bird atlas projects such as the atlas of breeding and winter birds of Great Britain and Ireland (Balmer et al. 2013), the atlas of common breeding birds of Poland (Kuczyński and Chylarecki 2012) and the Bird Atlas of the Netherlands (Sovon Vogelonderzoek Nederland 2018). According to a recent review, the random forests is the most commonly machine learning method in landscape ecology research (Stupariu et al. 2022).

The relative density maps produced with random forests can be converted into absolute density maps, showing the density expressed in numbers of birds. This is done by first calculating the current numbers of a species in the Netherlands by extrapolating numbers from the most recent Durch atlas data (Sovon Vogelonderzoek Nederland 2018) to the present on the basis of the trend of the species, annually calculated by the CBS Dutch Central Bureau of Statistic. Relative numbers per grid cell are then converted into absolute numbers in the relative density map.

For use in this project the 250 x 250 m absolute density maps were aggregated to a scale of 5 x 5 km.

## References

Bellin N., Tesi G., Marchesani N., Rossi V., 2022. Species distribution modeling and machine learning in assessing the potential distribution of freshwater zooplankton in Northern Italy. Ecological Informatics 69, 101682 <https://doi.org/10.1016/j.ecoinf.2022.101682>

Benito Garzon M., Blazek R., Neteler M., Sanchez De Dios R., Sainz Ollero H. & Furlanello C. 2006. Predicting habitat suitability with machine learning models: The potential area of *Pinus* *sylvestris* L. in the Iberian Peninsula. Ecological Modelling 197:383-393. DOI <https://doi.org/10.1016/j.ecolmodel.2006.03.015>

Breiman L. 2001. Random forests. Machine Learning Journal 45:5-32. URL https://link.springer.com/article/10.1023/A:1010933404324

Breiman L., Friedman J., Olshen R.A., Stome C.J., 1984. Classification and Regression Trees. Chapman & Hall, New York.

Centraal Bureau voor de Statistiek (CBS), 2012. Landelijke natuurmeetnetten van het NEM in

2011. Kwaliteitsrapportage NEM. CBS, Voorburg /Heerlen. URL <https://www.cbs.nl/nl-nl/publicatie/2012/12/meetprogramma-s-voor-flora-en-fauna-kwaliteitsrapportage-nem-over-2011>

de Jong A., Hustings F., Troost G., Bos G., van Winden E., van Els P., 2023. Handleiding LiveAtlas. Sovon Vogelonderzoek Nederland, Nijmegen. URL <https://pub.sovon.nl/pub/publicatie/20710>

Ebrahimi E., Araújo M.B., Naimi B., 2023. Flood susceptibility mapping to improve models of species distributions. Ecological Indicators 157, 111250. <https://doi.org/10.1016/j.ecolind.2023.111250>

Hengl T., Sierdsema H., Radovic A., Dilo A., 2009. Spatial prediction of species’ distributions from occurrence-only records: combining point pattern analysis, ENFA and regression-kriging.

Ecological Modelling 220, 3499-3511. DOI <https://doi.org/10.1016/j.ecolmodel.2009.06.038>

Hornman M., Hustings F., van Roomen M., Koffijberg K., van Winden E., Soldaat L., 2012. Populatietrends van overwinterende en doortrekkende watervogels in Nederland in 1975-2010. Limosa 85: 97-116. URL <https://pub.sovon.nl/static/publicaties/Limosa_85-3_2012_97-116Hornman_etal.pdf>

Hornman M., Koffijberg K., Louwe Kooijmans J., 2023. Handleiding Sovon Watervogel- en Slaapplaatsmonitoring. Sovon Vogelonderzoek Nederland, Nijmegen. URL <https://pub.sovon.nl/pub/publicatie/21874>

Kampichler C., Wieland R., Calmé S., Weissenberger H. & Arriaga-Weiss S. 2010. Classification in conservation biology: A comparison of five machine-learning methods. Ecological Informatics 5:441-450. DOI <https://doi.org/10.1016/j.ecoinf.2010.06.003>

Mascaro J., Asner G.P., Knapp E.E., Kennedy-Bowdoin T., Martin R.E., Anderson C., Higgins M. & Chadwick K.D. 2014. A tale of two “forests”: random forest machine learning aids tropical forest carbon mapping. PLoS ONE 9:e85993, DOI <https://doi.org/10.1371/journal.pone.0085993>.

Ritskes T.M., Daamen W.P., 1987. Doelstelling en uitvoering Vierde Bosstatistiek. Nederlands bosbouwtijdschrift 59(4):79-83.

Sierdsema H., van Loon E.E., 2008. Filling the gaps: using count survey data to predict bird density distribution patterns and estimate population sizes. Revista Catalana d’ Ornitologia 24:88-99.

Sovon Vogelonderzoek Nederland, 2018. Vogelatlas van Nederland. Broedvogels, wintervogels en 40 jaar verandering. Kosmos Uitgevers, Utrecht/Antwerpen.

Stupariu M.-S., Cushman S.A., Pleşoianu A.I., Pătru-Stupariu I., Fürst C., 2022. Machine learning in landscape ecological analysis: a review of recent approaches. Landscape Ecology 37, 1227–1250.

https://doi.org/10.1007/s10980-021-01366-9

van Manen W., de Jong A., 2024. Handleiding Punt Transect Telling (PTT). Sovon Vogelonderzoek Nederland, Nijmegen. <https://pub.sovon.nl/pub/publicatie/20644>
